# Visualizing Research Trends and Identifying Hotspots of Traditional Chinese Medicine (TCM) Nursing Technology for Insomnia: A 18-Years Bibliometric Analysis of Web of Science Core Collection

**DOI:** 10.3389/fneur.2022.816031

**Published:** 2022-03-31

**Authors:** Junxin Wang, Yufeng Chen, Xing Zhai, Yupeng Chu, Xiangdi Liu, Xueling Ma

**Affiliations:** ^1^School of Nursing, Beijing University of Chinese Medicine, Beijing, China; ^2^School of Traditional Chinese Medicine, Beijing University of Chinese Medicine, Beijing, China; ^3^School of Management, Beijing University of Chinese Medicine, Beijing, China

**Keywords:** insomnia, TCM, nursing, technique, visualization analysis, bibliometric

## Abstract

**Objective:**

To explore the research hotspot and frontier direction of TCM nursing of insomnia and provide reference for the follow-up study of the optimal scheme of TCM nursing of insomnia.

**Background:**

Insomnia is a common sleep-wake disorder, affects 6–10% of adults and was associated with independent higher risks of cardiovascular disease and diabetes. TCM Nursing Techniques of insomnia has a long history and has shown a definite impact. However, it's still lack of analysis in the field of the most commonly used and effective techniques, as well as the co-morbidities associated with insomnia. Therefore, the database was searched and analyzed to find effective TCM Nursing Techniques for insomnia and related diseases related to insomnia.

**Method:**

Randomized controlled trials on the intervention of TCM Nursing Techniques in insomnia were retrieved from Web of Science Core Collection and imported into CiteSpace 5.6.R5 visualization software. The works of literature were co-cited by keywords authors and institutions for visual analysis, and the co-morbidities associated with insomnia of TCM Nursing Techniques in literature was extracted manually. The symptoms of co-morbidities associated with insomnia were imported into Cytoscape 3.9.0 software and clustered by CytoHubba.

**Result:**

As of October 20, 2021, the literature published in the last 20 years from Web of Science Core Collection was screened, and the publication period of the included literature was from 2004 to 2021. From 2016 to now, the total number of articles has been increasing. A total of 146 articles were included, and the highest production year was 2020. There is little cooperation between states, institutions, and authors. China (including Hong Kong and Taiwan) and Hong Kong Polytech University are leading countries and institutions in this area. MYUNGHAENG HUR is the most cited author, and J ALTERN COMPLEM MED is the most cited journal. According to cluster analysis and keyword frequency, auricular therapy, aromatherapy, and acupressure are the three most commonly used techniques. While the top five co-morbidities are fatigue, anxiety, depression, pain and hemodialysis. The three frontier topics and the main research directions are sleep quality, comorbid insomnia and clinical trial design.

**Conclusion:**

We found that acupressure, aromatherapy, and auricular acupoint therapy are the most commonly used nursing methods of TCM to intervene in insomnia. However, these studies have limitations such as small sample size, lack of objectivity in evaluating sleep quality, and high heterogeneity of intervention measures, which are not conducive to forming TCM clinical nursing guidelines. Therefore, it is necessary to adopt objectified sleep quality evaluation methods, select suitable acupoints according to TCM theories, and design multi-center large-sample clinical trials based on the safety principle of randomized blind control. This study provides an in-depth perspective for insomnia research on TCM Nursing Techniques and includes information for follow-up research on TCM Nursing Techniques of insomnia.

## Introduction

In clinical practice, insomnia is one of the most common sleep disorders. The treatment of insomnia was originally mentioned in ancient Chinese medical books in “Nanjing” (Yellow Emperor's Classic of Eighty-one Difficult Issues). Chinese medicine calls it “Bu Mei,” “Bu De Mian,” “Bu De Wo” and so on. According to epidemiological surveys conducted around the world, the population diagnosed with insomnia accounts for 6 to 10% of the total population (1). Insomnia is becoming more common as social stress levels rise (2). According to subjective evaluation criteria, the percentage of individuals who fit the diagnostic criteria for insomnia ranges from 10 to 15%, and current research indicates that insomnia is linked to mental problems and physical ailments in a variety of ways (3). Insomnia reduces patients' learning and work efficiency, increases error rates, harms patients' quality of life, and significantly burdens society. As a result, their quality of life suffers greatly, and develops distressing and frustrating feelings. In recent years, there has been the concept of comorbid insomnia (4), insomnia combined with endocrine diseases, cardiovascular diseases and other diseases of patients began to increase. Therefore, we hope to explore the field of comorbid insomnia (5, 6).

There are numerous studies of insomnia in recent years, however owing to the subjective nature of insomnia, there are still limits in definitions, quantitative standards, epidemiological investigations, and other elements of insomnia (7–9). International specialists are still actively seeking effective treatments for insomnia. Many nations' guidelines (10–12) separate adult chronic insomnia treatment into two categories: Pharmacotherapy and non-pharmacotherapy. Pharmacotherapy is usually administered with sedative-hypnotics such as benzodiazepines. However, these therapies have various drawbacks, such as side effects, patients' dissatisfied attitudes, and contraindications (13–15). Non-pharmacological treatments include cognitive behavioral therapy for insomnia (CBT-I). Although CBT-I is currently considered the most effective non-pharmacological treatment for insomnia, CBT-I has sleep restriction and other demanding treatments (16). The treatment may be the factor in the stress experienced by patients during CBT-I, directly or indirectly leading to increased anxiety and depressive symptoms during treatment (17). Because the need for western therapy to control insomnia has not been met, medical staffs began to consider the possible role of TCM in preventing and treating insomnia, and a number of clinical studies in this field are attracting more and more attention in the sleep field.

TCM nursing is a subject which is old yet young. Guided by theories of TCM, it adopts the concept of holism and nursing determination based on pattern differentiation. TCM nursing has rich connotation and unique theories, approaches and techniques. It is a practical science, which combines prevention, health care, rehabilitation and medicine to take care of the sick, the old, the weak, the young and the handicapped, and implements specific nursing techniques to safeguard the health of people (18). Meanwhile, TCM Nursing Techniques is also an essential part of Chinese medicine, is the application of Chinese medicine therapy in clinical nursing work and expands the scope of care. TCM Nursing Techniques has the advantages of being simple, convenient, effective, and low cost sincerely welcomed and favored by the people (19). TCM Nursing Techniques gives play to the holistic concept of traditional Chinese medicine and the characteristics of comprehensive dialectical regulation in treatment to overcome the single blocking sedation treatment phenomenon. More and more clinical trials as evidence support the effectiveness of TCM Nursing Techniques in the treatment of insomnia (20–23). TCM Nursing Techniques are widely used in the clinic and are highly accepted by patients. In 2016, China published its first TCM clinical practice guidelines for insomnia—Guidelines for clinical practice of Chinese medicine in insomnia (WHO/WPO) (24), and the TCM Diagnosis and Treatment Program of Insomnia (25) were updated in the same year. The holistic concept of Traditional Chinese Medicine has made outstanding contributions to the research of life sciences. The use of Traditional Chinese Medicine to treat insomnia has improved the prevention and treatment level of insomnia in the world and played an essential role in reducing the economic burden of health. The above two guidelines combine the internationally recognized diagnostic standards and treatment norms of insomnia and summarize the current evidence-based medical evidence of TCM (including TCM Nursing Techniques) in preventing and treating insomnia. Compared with traditional Chinese medicine formula, TCM Nursing Techniques avoids the side effects such as liver and kidney damage that may be caused by oral administration. However, both guidelines point out that TCM treatment of insomnia still needs further improvement. In recent years, researchers have conducted many studies on TCM Nursing Techniques in treating insomnia, and many research results have been published worldwide. The field of TCM nursing is undergoing a period of rapid development. However, no comprehensive analysis has been undertaken to describe the distribution and contribution of the research output of global institutions. Therefore, we conducted a bibliometric analysis to comprehensively understand the research trend of TCM nursing techniques in insomnia intervention from multiple aspects. Our research objective is to provide a valuable reference for clinical researchers and practitioners.

## Materials and Methods

### Data Collection

Data for this article were collected from the Web of Science Core Collection Database, October 20, 2021. The search strategy consists of three parts. First, we have listed the indexes of various TCM Nursing Techniques. The Scope of TCM techniques were refered to the National Administration of Traditional Chinese Medicine (SATCM), which is a national standard, including 18 TCM techniques (26). The leading 21 retrieval words were #1 TCM nursing technology, #2 moxibustion, #3 auricular therapy, #4 cupping, #5 Acupoint Application, #6 acupoint injection, #7 Acupressure, #8 Scraping Therapy, #9 Medical Wax Therapy, #10 Foot Bath Therapy, #11 cold compress therapy, #12 Wet Compress Therapy, #13 Drug Smearing Therapy, #14 Fumigating and Steaming Therapy, #15 Chinese medicine hot pressing compress, #16 Chinese Medicine Ion Introduction Therapy, #17 Retention Enema with Chinese Herb. These words are translation The search comprises keywords and extensions, covering any time, English languages and all document types.

Second, subject searches focus on index terms related to insomnia. Search strategy = (insomnia) OR (sleeplessness) OR (sleep initiation) OR (maintenance disorders) OR (disorders of initiating and maintaining sleep) OR (primary insomnia) OR (transient insomnia) OR (secondary insomnia) OR (insomnia disorder) OR (sleep initiation dysfunction) OR (quality of sleep) OR (sleep complaint) OR (sleep problem) OR (sleep disturbance) OR (sleep disorder). The language document type and period were the same as the first query, which produced 134,978 records.

Third, because there are more than 100 words in the search strategy, the Web of Science database cannot recognize it, so we split the search strategy into five parts, respectively combined with the insomnia search strategy.

Finally, we screened out literature on TCM Nursing Techniques for insomnia from the retrieval results of five parts, selected clinical trials, included and excluded the scientific achievements of conference reports, newspapers, and expert experience, and obtained 146 records in total. The exported file is in the download_^***^. TXT format that CiteSpace can recognize. The imported data is complete records and referenced references. The subject search query is shown in Appendix 1, 2 in Supplementary Material.

### Analysis Tool

#### Data Management Bibliometric Analysis

CiteSpace 5.6.R5 used a visual knowledge graph to discover the research frontiers and emerging trends in TCM Nursing Techniques for insomnia. The following parameters were set: (1) Time period (2001–2021), 1 year per slice; (2) Pruning pick pathfinder and pruning sliced networks, Top *N* = 25. Aside from that, all other settings are set to default; (3) Cluster analysis was performed using “K” and “LLR.” Modularity Q and Mean Silhouette were employed in the investigation and assessment of the cluster map. Generally, clustering is substantial and feasible when Q > 0.3 and S > 0.5. Meanwhile, S > 0.7 indicates that the grouping is convincing. The development trend and research hotspots in the field of TCM nursing of insomnia can be intuitively presented by drawing the multi-time-sharing dynamic visualization knowledge map and the change of publication quantity over time (27).

#### Cluster Analysis Based on Article Content

Two researchers extracted the information of 146 articles, and the TCM Nursing Techniques, co-morbidities associated with insomnia, and symptoms of co-morbidities associated with insomnia were extract from the articles, respectively and sorted by Excel (Microsoft Office Home and Student 2019). Finally, we obtained 14 TCM Nursing Techniques, 69 co-morbidities associated with insomnia, and 6 main symptoms of co-morbidities associated with insomnia. The sorted Excel table was imported into Cytoscape 3.9.0 for mapping. CytoHubba plug-in was used for clustering with Degree algorithm (the higher Degree value is, the more connections the node has).

## Results

### Trends in Research Publications

**Figure 1:**
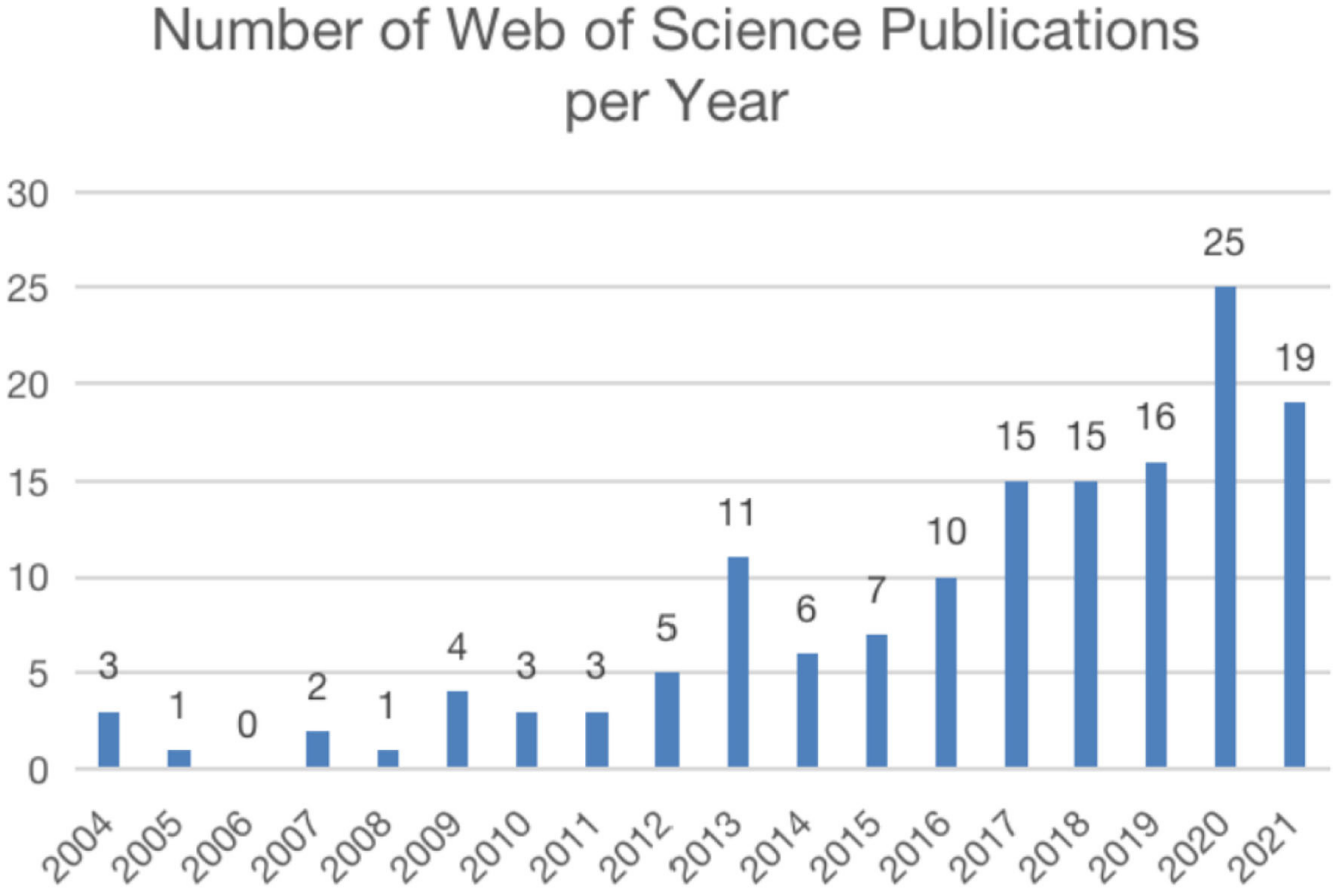
Number of publications per year. The annual number of publication on acupuncture in insomnia.

According to the retrieval, the clinical study on TCM Nursing Techniques for insomnia was first published in 2004, so we conducted a comprehensive analysis of the global situation of the research results from 2004 to 2021. The included literature were plotted into a bar chart, and the results showed that the overall number of articles presented a steady upward trend. According to Figure 1, we can find the research trends in several stages. The first stage was 2004 and 2012, which was the trough period of clinical research of TCM Nursing Techniques to intervene insomnia. The overall number of articles published fluctuated in a small range and presented a stable trend, with about three articles published every year, but the number of articles published in 2006 was 0. In the second stage, 2013 saw a rapid growth in the number of articles published. A total of 11 articles were published, which was the year with the largest number of articles published in the first decade. The third stage, from 2014 to 2021, saw rapid growth in the number of publications. Although the number of articles published in 2014 and 2015 decreased slightly compared with 2013, it began to recover and proliferate in 2016, especially in 2020, the number of articles published reached 25.

### Analysis of Countries and Institutes

**Figure 2:**
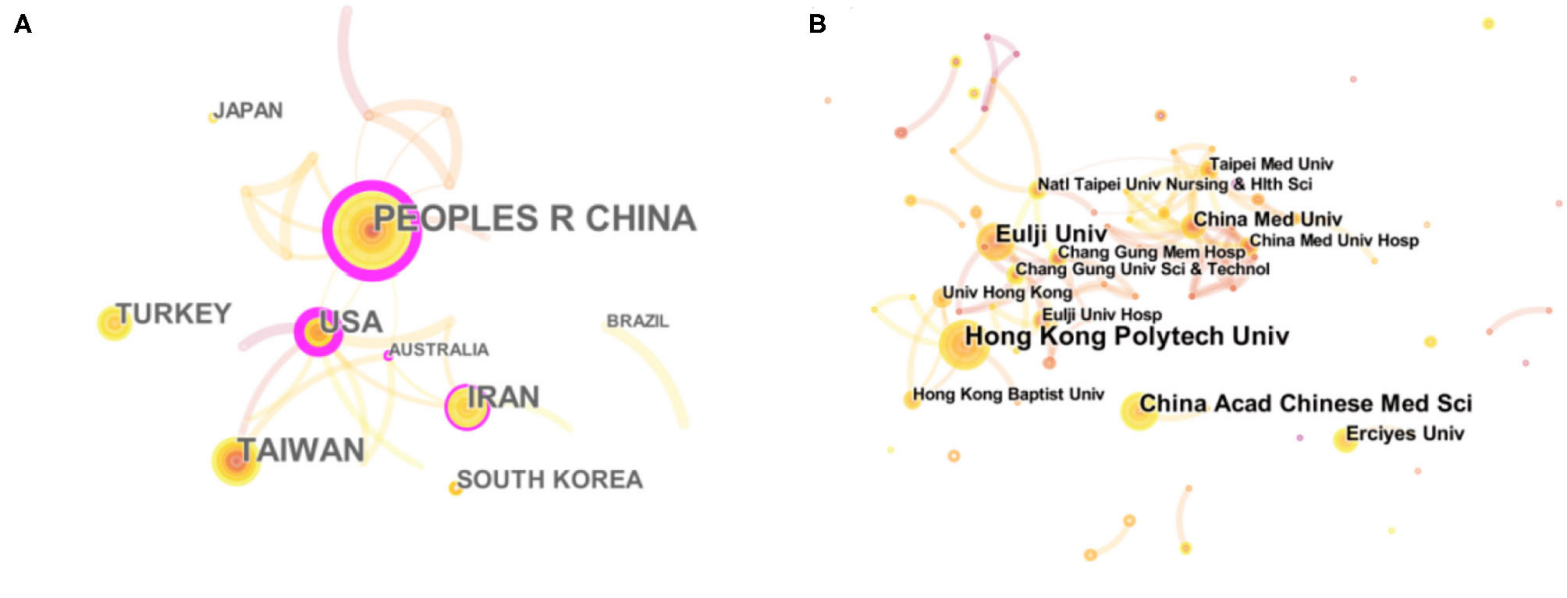
Network map showing the relations between various countries **(A)** and the Institutions **(B)**.

Twenty-eight different countries or regions have contributed to this literature. Table 1 lists the top five countries in terms of the number of publications and centrality. The national network map is represented by 28 nodes and 30 links (Figure 2A). China (including Taiwan), as the origin of TCM Nursing Techniques, published 49 out of 146 articles, accounting for 33.56% of the included articles. Meanwhile, the United States and Iran ranked third and fourth in terms of the number of publications, respectively, with frequency of 20, indicating that TCM Nursing Techniques in treating insomnia have attracted much attention in these two countries. The top five countries were China (0.57), USA (0.45), Australia (0.12), Iran (0.11), and England (0.1), all of which centrality were more than 0.1, indicating that these five countries had sound mediating effects (Table 1).

**Table 1:** Top 5 country/region and institute.

**Rank**	**Country/region**	**Frequency**	**Country/region**	**Centrality**	**Institution**	**Frequency**
1	PEOPLES R CHINA	33	PEOPLES R CHINA	0.57	Hong Kong Polytech Univ	7
2	TAIWAN	26	USA	0.45	China Acad Chinese Med Sci	6
3	USA	20	AUSTRALIA	0.12	Eulji Univ	6
4	IRAN	20	IRAN	0.11	China Med Univ	4
5	TURKEY	17	ENGLAND	0.10	Erciyes Univ	4

The Hong Kong Polytechnic University (PolyU) tops the list of research institutes studying TCM Nursing Techniques for insomnia (Figure 2B), followed by the China Academy of Chinese Medical Sciences. In addition, Eulji University in South Korea, China Medical University and Erciyes University in Turkey have carried out many studies in the field (Table 1). However, auricular therapy is the most studied TCM Nursing Techniques in the Hong Kong Polytechnic University (28, 29), China Academy of Chinese Medical Sciences (30, 31), and China Medical University (32, 33), followed by acupressure. Aromatherapy is the primary TCM Nursing Techniques studied by institutions from Korea (34, 35) and Turkey (36, 37).

### Journal Distribution

**Figure 3:**
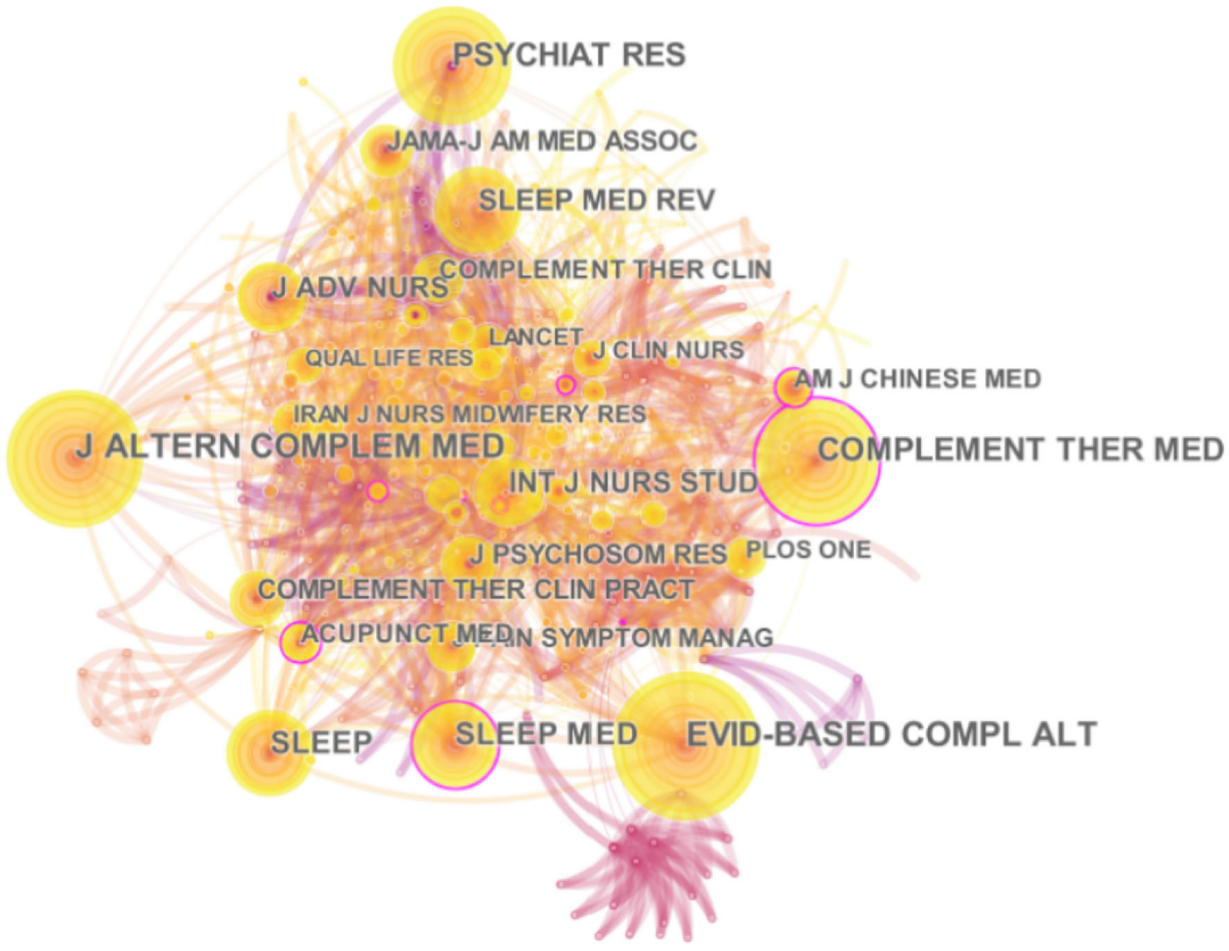
Network map of cited journal.

This study uses the CiteSpace system to generate a cited journal map with node 408 and line 1829 (Figure 3). J ALTERN COMPLEM MED (IF 2020 = 2.582) is the most-cited journal in the research field of TCM Nursing Techniques for insomnia, and the second cited journal is EVID-BASED COMPL ALT (IF 2020 = 2.63) (Table 2). Interestingly, the top five journals in citation frequency and centrality were all different. The journal with the highest centrality was AM J CHINESE MED (IF 2020 = 4.667), and the most frequently cited article on sleep in the journal was a randomized controlled trial published by Italian researchers, which was cited 77 times in total. Studies have shown that at the end of 6 months of acupressure treatment for adolescent insomnia, the intervention group's sleep efficiency and sleep duration were significantly improved. This study concluded that acupressure treatment for adolescent insomnia is a non-invasive, safe, and effective treatment with good compliance and less adverse reactions (38).

**Table 2:** Top 5 cited journal.

**Rank**	**Cited journal**	**Frequency**	**Cited journal**	**Centrality**
1	J ALTERN COMPLEM MED	68	AM J CHINESE MED	0.22
2	EVID-BASED COMPL ALT	67	ACTA PSYCHIAT SCAND	0.20
3	COMPLEMENT THER MED	59	SLEEP MED	0.17
4	PSYCHIAT RES	58	ANN INTERN MED	0.17
5	SLEEP	43	BRIT MED J	0.16

**Figure 4:**
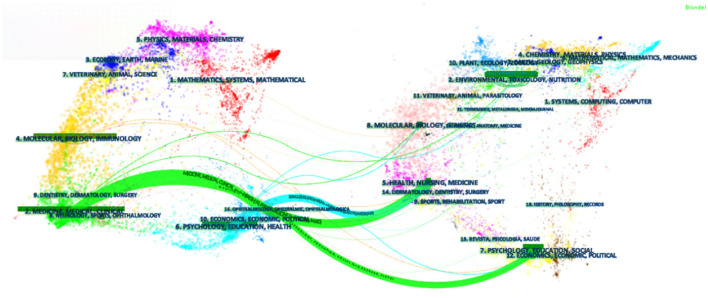
The dual-map overlay of journals related to PE research. [The Dual-map Overlay of Journals Related to the treatment of Insomnia by TCM Nursing Techniques research. There were three citation paths. The top green path, articles published in medicine/medical/clinical journals mostly cited journals in health/nursing/medicine area; the bottom green path, articles published in medicine/medical/clinical journals partially cited journals in psychology/education/social area. The blue path, articles published in psychology/education/health journals partially cited journals in health/nursing/medicine area. (Color figure online)].

Figure 4 shows the double map coverage of journals similar to the cited and cited journal maps on the left and right, respectively. Each covered journal field type is represented by a label indicating the citation status of each journal. From left to right, green and blue lines show the double map overlay showing the three main citation paths for this topic.

### Analysis of Authors

**Figure 5:**
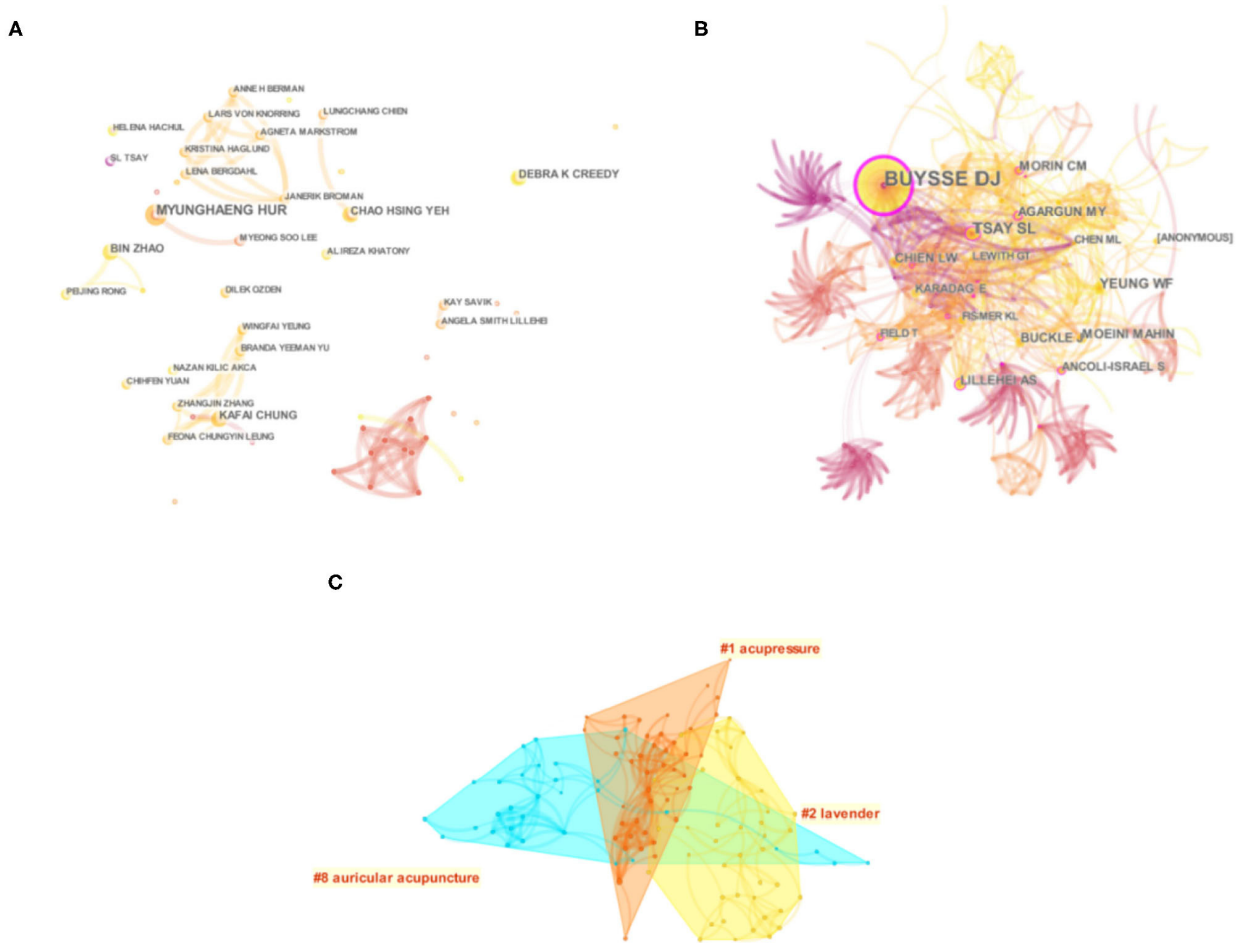
Network map of author **(A)**, cited author **(B)** and cluster map of cited author **(C)**.

Among the top five authors cited frequently (Figure 5A; Table 3), MYUNGHAENG HUR from South Korea ranked the first (34, 39–41), published four articles in total, mainly studying aromatherapy and the combination of aromatherapy and massage. The other four authors are Kuo et al. (42), Yeh et al. (43), Zhao et al. (44), and Bergdahl et al. (45). However, these authors are from different countries/regions and institutions (Hong Kong, Taiwan, and the United States) China). They are both active in researching the effects of auricular therapy, a TCM Nursing Techniques on insomnia. Although the authors ranked 2–4 have similar research fields, it can be seen from the author's co-occurrence network map that there is almost no cooperation among these authors.

**Table 3:** Top 5 authors and cited authors.

**Rank**	**Author**	**Count**	**Cited author**	**Count**	**Centrality**
1	MYUNGHAENG HUR	4	BUYSSE DJ	61	0.44
2	DEBRA K CREEDY	3	TSAY SL	19	0.16
3	CHAO HSING YEH	3	YEUNG WF	16	0.05
4	BIN ZHAO	3	LILLEHEI AS	14	0.15
5	JANERIK BROMAN	2	CHIEN LW	14	0.10

Author co-citation data was analyzed by CiteSpace and visualized by co-citation network diagram, forming a network diagram of cited authors with 330 nodes and 509 connections (Figure 5B). In the co-cited author atlas cluster, the main TCM Nursing Techniques for insomnia are acupressure, aromatherapy, and auricular therapy (Figure 5C). BUYSSE DJ topped the cited authors list (Table 3), followed by TSAY SL, YEUNG WF, LILLEHEI AS and CHIEN LW. BUYSSE DJ ranked first in co-citation frequency and centrality, with a co-citation frequency of 61 times. He is a sleep expert and developed the Pittsburgh Sleep Quality Index, which is still used today to assess sleep quality (46).

### Analysis of References and Cocitations

**Figure 6:**
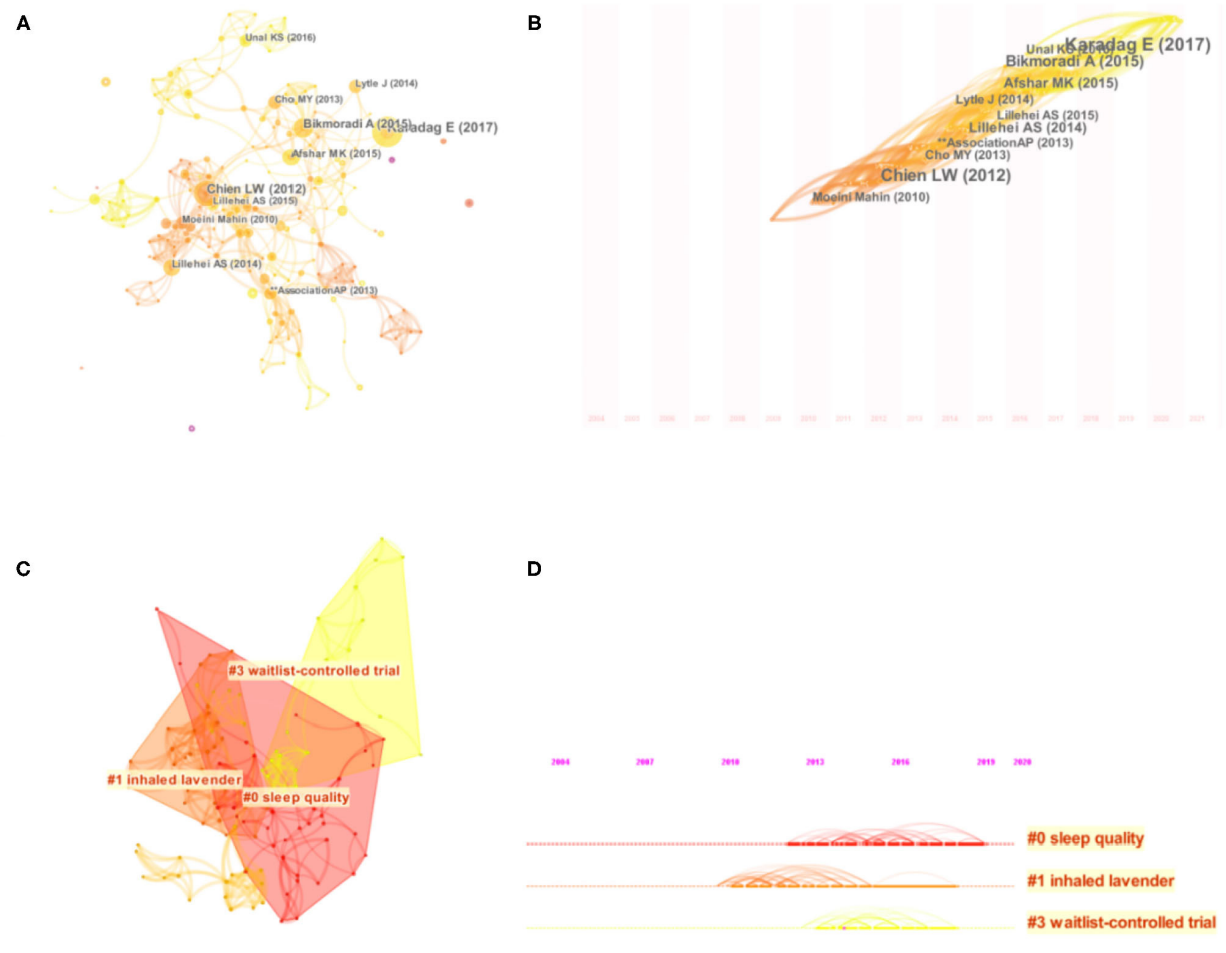
Network map **(A)**, timezone view **(B)**, cluster map **(C)** and timeline **(D)** of the cocitation reference.

The co-cited literature network map consists of 419 nodes and 1,140 connections (Figures 6A,B). Based on the clustering of citation keywords, the clustering module value (Modularity, Q value) is 0.9271 (>0.3), the community structure in the cluster is significant, with an average Silhouette (S value) of 0.6722 (>0.7), the clustering can be convincing.

Table 4 lists the top five most-cited articles from 2004 to 2021. In terms of citation frequency, Karadag (56) article published in 2017 ranks the first, which is still actively cited up to now. A randomized controlled study using aromatherapy (lavender essential oil) in 60 patients in a southeastern Turkish province improved sleep quality in the intensive care unit (ICU). It recommended the use of cost-effective independent nursing interventions for cardiac patients in the ICU. The top five articles were all studies on aromatherapy for insomnia. One was a systematic review and the rest were clinical trials.

**Table 4:** Top 5 cited references.

**Rank**	**Cited reference**	**Frequency**	**Title (Country/Region)**	**Cited reference**	**Centrality**	**Title** **(country/region)**
1	Karadag (47)	9	Effects of aromatherapy on sleep quality and anxiety of patients DOI 10.1111/nicc.12198 (Turkey)	Ancoli-Israel (48)	0.12	Sleep, fatigue, depression, and circadian activity rhythms in women with breast cancer before and after treatment: a 1-year longitudinal study DOI 10.1007/s00520-014-2204-5 (USA)
2	Chien (49)	8	The Effect of Lavender Aromatherapy on Autonomic Nervous System in Midlife Women with Insomnia DOI 10.1155/2012/740813 (Taiwan)	Afshar (50)	0.11	The effects of guided imagery on state and trait anxiety and sleep quality among patients receiving hemodialysis: a randomized controlled trial DOI 10.1016/j.ctim.2018.07.006 (Iran)
3	Bikmoradi (51)	6	Effect of inhalation aromatherapy with lavender essential oil on stress and vital signs in patients undergoing coronary artery bypass surgery: A single-blinded randomized clinical trial DOI 10.1016/j.ctim.2014.12.001 (Iran)	Arai (52)	0.10	Auricular Acupuncture at the “Shenmen” and “Point Zero” Points Induced Parasympathetic Activation DOI 10.1155/2013/945063 (Japan)
4	Lillehei (53)	5	A Systematic Review of the Effect of Inhaled Essential Oils on Sleep DOI 10.1089/acm.2013.0311 (USA)	Lan (54)	0.09	Auricular acupuncture with seed or pellet attachments for primary insomnia: a systematic review and meta-analysis DOI 10.1186/s12906-015-0606-7 (China)
5	Afshar (55)	5	Lavender Fragrance Essential Oil and the Quality of Sleep in Postpartum Women DOI 10.5812/ircmj.17(4)2015.25880 (Iran)	Chien (49)	0.08	The Effect of Lavender Aromatherapy on Autonomic Nervous System in Midlife Women with Insomnia DOI 10.1155/2012/740813 (Taiwan)

Ancoli-israel Sonia's article (48) was not only the most co-cited in mediating centrality, with a centrality of 0.12(>0.1) (Table 4), and cited 97 times, so this article has a high impact. The results of this controlled longitudinal study showed that breast cancer patients still had poorer sleep quality than non-cancer patients even one year after treatment. However, further research is needed to determine whether treatment of sleep disorders prior to initiation of chemotherapy minimizes the severity of symptoms over time, and the timing entry points for various interventions are tentatively explored.

In order to obtain critical clusters for citation, representative clusters are selected from clusters generated on the network atlas to reflect research patterns and emerging trends (Figure 6C; Table 5). The largest cluster is Sleep Quality, with a Silhouette value of 0.848, indicating that the results are meaningful. The second cluster is inhaled lavender, with a Silhouette value of 0.919. The third cluster, WaitList-Controlled trial, has a Silhouette value of 0.927. As can be seen from the timeline (Figure 6D), the Waitlist-Controlled trial has a high centrality. Randomized controlled trials (RCTs), the most commonly used method to study clinical efficacy, must be carefully designed. The establishment of the waiting group, to some extent, avoids the placebo effect, meets the ethical requirements, and increases the credibility of research results.

**Table 5:** Clusters in the reference cooccurrence network.

**ID**	**Cluster**	**Size**	**Silhouette**	**Mean(Year)**
0	Sleep quality	40	0.848	2015
1	Inhaled lavender	34	0.919	2012
3	Waitlist-controlled trial	22	0.927	2015

### Analysis of Keyword

**Figure 7:**
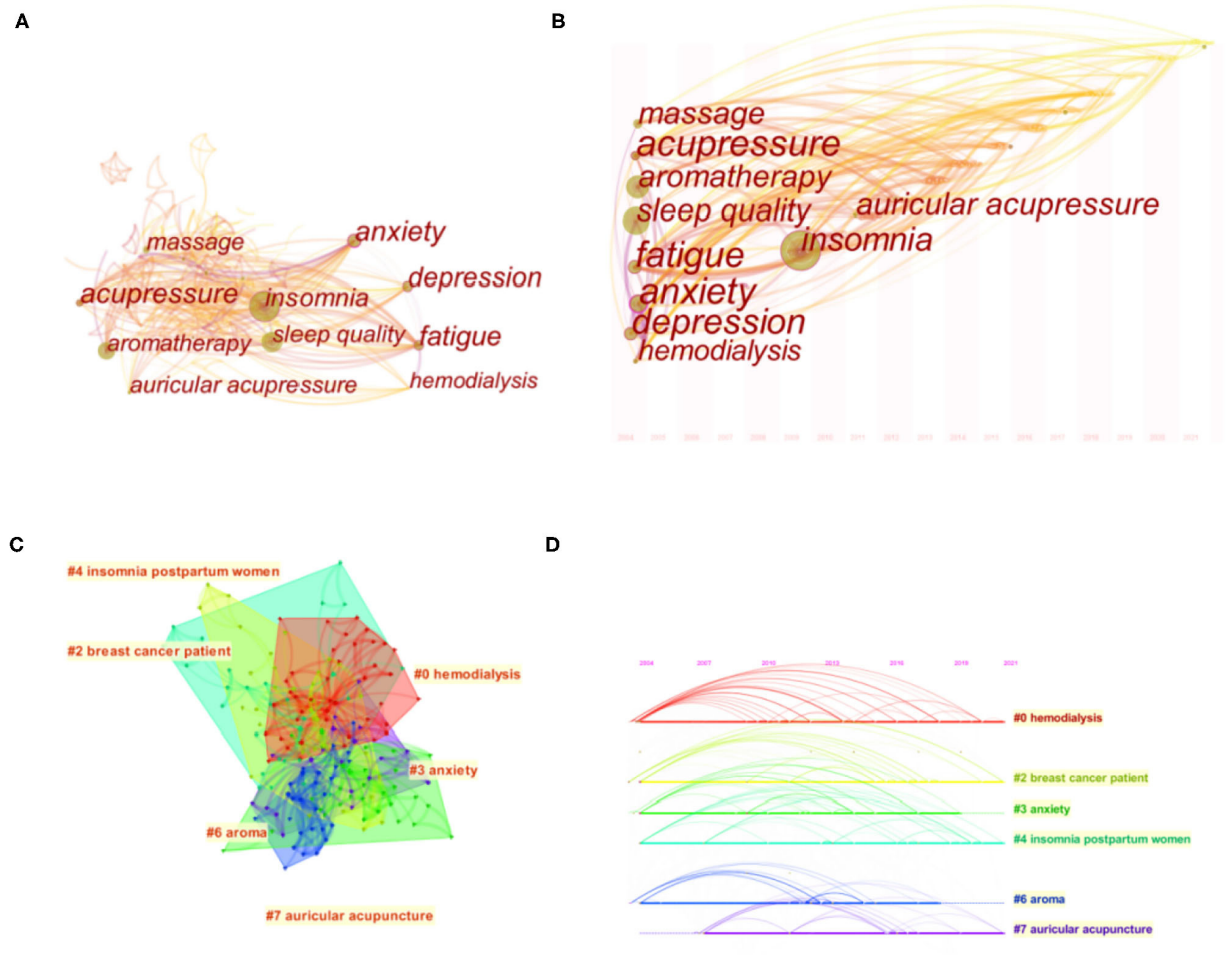
Network map **(A)**, timezone view **(B)**, cluster map **(C)** and timeline **(D)** of the keywords.

Keywords co-occurrence can effectively reflect research hotspots and cutting-edge topics and provide research support. Insomnia can occur not only alone but in combination with other conditions. From 286 notes and 1069 links through keyword co-occurrence graph (Figure 7A), the top five of co-morbidities associated with insomnia is fatigue, anxiety, depression, pain, and hemodialysis. The frequency and centrality of anxiety were relatively high. The top five TCM Nursing Techniques for insomnia is aromatherapy, acupressure, massage, auricular acupressure and aromatherapy massage. The frequency and centrality are relatively high TCM Nursing Techniques is aromatherapy (Tables 6, 7). As can be seen from the timezone view (Figure 7B), in recent years, more and more researchers have begun to pay attention to auricular therapy to treat sleep disorders associated with various diseases, such as hemodialysis and hypertension (57, 58). In particular, hemodialysis became the most significant cluster and has been active since 2012 (Figures 7C,D).

**Table 6:** Top 10 key words in terms of frequency and centrality.

**Rank**	**Keyword**	**Count**	**Keyword**	**Centrality**
1	Insomnia	46	Anxiety	0.2
2	Aromatherapy	33	Fatigue	0.19
3	Sleep quality	32	Acupressure	0.16
4	Fatigue	27	Sleep	0.16
5	Anxiety	26	Insomnia	0.14
6	Depression	22	Care	0.14
7	Acupressure	21	Depression	0.12
8	Sleep	20	Disorder	0.1
9	Therapy	17	Massage	0.09
10	Massage	15	Therapy	0.08

**Table 7:** Top 5 TCM nursing technology treatment and insomnia comorbidity in terms of frequency and centrality.

**Rank**	**TCM nursing technology**	**Count**	**Centrality**	**Comorbidity**	**Count**	**Centrality**
1	Aromatherapy	33	0.06	Fatigue	27	0.19
2	Acupressure	21	0.16	Anxiety	26	0.2
3	Massage	15	0.09	Depression	22	0.12
4	Auricular acupressure	9	0.05	Pain	11	0.03
5	Aromatherapy massage	4	0.01	Hemodialysis	7	0.05

### Clustering Based on Article Content

**Figure 8:**
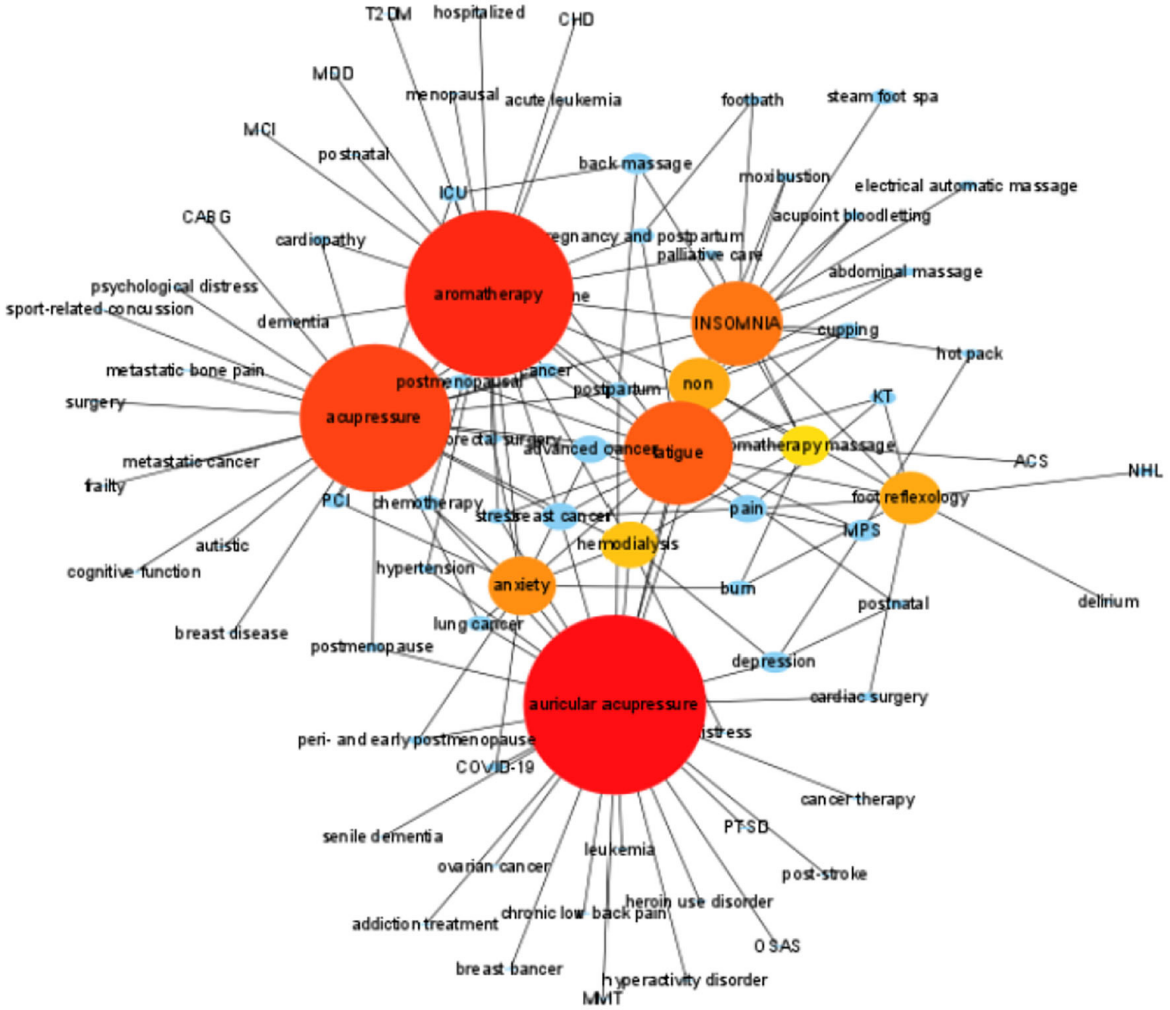
Clustering graph based on article content.

Using Cytoscape3.9.0, the clustering diagram based on Degree value is obtained. The results show that the top 10 content are auricular acupressure, aromatherapy, acupressure, fatigue, INSOMNIA, anxiety, non (insomnia without co-morbidities), foot reflexology, hemodialysis, aromatherapy massage. With these several contents as the center, formed several main modules (Figure 8).

## Discussion

### General Overview

The mechanism of insomnia is very complex, including the central and peripheral nervous systems and the endocrine system. So far, there is still no clear consensus on the pathogenesis of insomnia in modern medicine. Still, it is generally believed that pathogenesis weakens inhibitory function or enhances awakening function during the sleep-wake cycle (59). The understanding of TCM about the pathogenesis of insomnia is the imbalance of Yin and Yang, involving the disharmony of zang-fu and the disharmony between Ying and Wei, which is similar to the general understanding of Western medicine (60). In the theory of TCM, the meridians and collaterals run qi and blood to connect the zang-fu organs with the body surface and all parts of the body. Qi is divided into Yin and Yang. The movement of qi rising and falling in and out promotes and regulates human life activities, and qi impassability leads to the occurrence and development of diseases. Insomnia is associated with many aspects of mental disorders and physical diseases. Comorbid insomnia has received extensive attention from researchers.

Complementary and alternative medicine (CAM) has been used in insomnia worldwide, and although TCM Nursing Techniques are now included in the recommendations of sleep guidelines, there is still a lack of high-quality research and evidence-based evidence to support this treatment. There is limited evidence that TCM Nursing Techniques can improve insomnia symptoms. A systematic review of 40 randomized controlled trials concluded that acupressure, reflexology, or auricular massage, which places physical pressure on acupoints or reflex areas, performed significantly better alone or in combination with conventional care than with or without conventional care (61). As a widely used, relatively safe, and widely accepted intervention, TCM Nursing Techniques can improve sleep quality and have no adverse effects on insomnia.

In this study, Cite Space 5.6.R5 was used to conduct statistical analysis of relevant literature published in 2004 and 2021 on TCM Nursing Techniques in the treatment of insomnia, including 146 articles. As can be seen from the publications about insomnia research on TCM Nursing Techniques, it has been presented a fluctuating upward trend, and the number of publications has increased significantly in the last few years. This phenomenon may be related to the development of Complementary and Alternative Medicine. It indicates that there is a great potential in the future.

### Analysis of Countries, Research Institutions and Published Journals

The output countries are mainly from the China and USA (Table 2). China published the most articles, suggesting that TCM Nursing Techniques is widely accepted in China. Moreover, since the centrality of China is the highest, it is also the central collaborator of other countries. The output institutions are mainly from the University of Hong Kong, Beijing, Korea, Shenyang and Turkey. In addition, China and its three institutions occupy a central position and maintain a high degree of cooperation with countries and institutions. Collaboration helps researchers who investigated insomnia research on TCM Nursing Techniques share resources and exchange knowledge and ideas, which is crucial for further development of the insomnia research on TCM Nursing Techniques. Thus, stronger collaboration networks should be established among more countries, institutes, and authors, especially in China. However, there is not yet an excellent cooperative relationship between countries and institutions. Although there are more than 20 TCM universities in China, the research on TCM Nursing Techniques for insomnia intervention is still in its infancy, which may be related to the late establishment of TCM nursing disciplines and the more emphasis on clinical efficacy. The top five authors are from CAM or insomnia backgrounds. Although fewer authors were at individual nodes in the Web of Science database, there was little collaboration between the teams, indicating that no research team has been established to study auricular therapy for insomnia. It indicates poor communication and cooperation awareness among scholars worldwide in studying TCM appropriate technology intervention in insomnia. The academic exchange and collaboration between scholars need to be strengthened urgently. It is more important to enhance cooperation with experts with other professional backgrounds.

The top three journals are Journal of Alternative and Complementary Medicine, Evidence-based Complementary and Alternative Medicine and Complementary Therapies in Medicine. This indicated that TCM Nursing Techniques is regarded as complementary and alternative medicine. Although it is challenging to publish articles related to TCM Nursing Techniques in high-impact factor journals, it can be seen from the trend of publication volume that more research on TCM Nursing Techniques in the treatment of insomnia has been in progress in recent years. Current bibliometric analysis shows that more and more experts from different disciplines (such as hemodialysis, anxiety, etc.) are studying this area, and many clinical studies have been published so far. Research with good design and quality control should be published in high-quality journals gradually, which is conducive to exchange of research findings on insomnia research on TCM Nursing Techniques.

### Research Hot Spots and Trends

Keywords show the researchers' research results and help to understand the research progress. We combined keywords and cluster graph analysis to reveal the research hot spots and development trends in TCM Nursing Techniques of insomnia. From the keyword network graph, anxiety has a high frequency and ranks first in the centrality. Among the symptoms of co-morbidities associated with insomnia, 73% of anxiety occurs before insomnia, and 69% of the comorbidity cases of insomnia and depression occur before insomnia (62). In addition, anxiety levels were 17.35 times higher in insomniacs than in non-insomniacs, and increased insomnia frequency was associated with increased depression and anxiety (63). Our results reaffirm the close relationship between insomnia and anxiety and highlight the clinical predictive role of insomnia in future anxiety and/or depressive episodes (64).

Our cluster analysis of keywords reveals two critical research areas on the use of TCM Nursing Techniques to treat insomnia. This article mainly deals with the diseases currently studied, such as hemodialysis, breast cancer, anxiety, and postpartum women; and the commonly used TCM Nursing Techniques, such as Aromatherapy and Auricular therapy. And hemodialysis is the largest cluster. In our study, we deeply analyzed the articles of 5 authors with the most published literature and the most cited articles and found that the most common topics in the study mainly revolve around auricular therapy, acupressure, and aromatherapy. In a word, the trend of insomnia research on TCM Nursing Techniques mainly focuses on TCM Nursing Techniques can improve the sleep quality of insomnia complicated with diseases, especially hemodialysis complicated with insomnia.

Among the cocitation reference, waitlist-controlled trials formed the third-largest cluster. Due to ethical limitations, it is impossible to select a physiologically similar placebo for comparison in clinical studies of TCM Nursing Techniques. The wait-list control group, as a baseline, gives researchers a good idea of how the subjects who got the intervention were different from the subjects who didn't get the intervention at the same time. There's always a debate about how to choose the proper control group.

### The Most Studied TCM Nursing Technique for Insomnia Intervention

#### Acupressure

In different types of insomnia, acupressure can significantly improve the quality of sleep loss with high safety (65). Since there is still no conclusion on the acupuncture points stimulated by acupressure in current studies, and HT7 acupoint of heart meridian as an important acupoint for insomnia has not been supported by sufficient high-quality evidence, more attention needs to be paid to this therapy (66).

#### Aromatherapy

It can be seen from the frequency and contribution chart of cited journals and researchers that aromatherapy for insomnia has been studied and applied more in countries outside China, which may be related to the deep TCM theory in TCM Nursing Techniques. As far as aromatherapy is concerned, it is a safe, inexpensive, and effective tool for improving insomnia symptoms in patients with very little training (67). Several meta-analyses of random-effect models have shown that the use of aromatherapy can alleviate sleep problems in patients with insomnia, especially early sleep problems (53, 56, 68, 69). Meta analysis shows that essential oil massage is more effective than essential oil inhalation in improving sleep quality (56). However, due to the high heterogeneity between studies, inferences from the results need to be applied with caution. More in-depth and detailed studies on the pharmacological activities of various essential oils used in aromatherapy are needed in the future. More scientific, rigorous, and strong trials are needed to analyze the actual effects of inhalation aromatherapy on sleep. Because of the clinical significance of sleep-affected wakefulness, measurements of wakefulness outcomes should also be considered. For people with mild sleep disorders, aromatherapy can be considered. Further studies with a larger sample will be needed to build on these findings. Therefore, it is essential to develop specific guidelines for effective inhalation aromatherapy.

#### Auricular Therapy Is the Most Potential Used TCM Nursing Technique to Intervene Insomnia

From the research of national institutions and the cluster and time zone view of keyword formation, we can infer that auricular acupoint therapy may be a better TCM Nursing Techniques for insomnia. Auricular therapy is the core discipline of traditional Chinese Medicine (TCM), which has been practiced for hundreds of years in China. Huangdi Neijing mentioned the ear is closely related to the five Zang-organs and six Fu-organs, and twelve regular channels all reach the ear directly or indirectly. So ancient Chinese medicine has noticed that people can use the auricle to diagnose diseases, especially the kidney disease.

First, the mechanism of auricular acupoint therapy is relatively clear. French doctor P Nogier first proposed the embryonic inverted auricular diagram, and carried out extensive practice and verification in clinical practice. In recent years, many studies have shown that part of the efficacy of auricular therapy is due to its stimulation of the vagus nerve in the ear. Local cutaneous electrical changes reflect skin motor activity controlled by the autonomic nervous system (ANS). Usichenko et al. summarized relevant clinical and experimental results and found that the anatomical structure of the external auricular nerve pathway is similar to the mechanism of action of percutaneous vagal nerve stimulation (70). Not only that, but transcutaneous vagal nerve stimulation also affects melatonin production. The study of transcutaneous vagus nerve stimulation (tVNS) is beneficial to enrich the evidence support of auricular therapy, thus contributing to the further development of auricular therapy (71). Moreover, the effect of Shenmen acupoint has been recognized. A systematic review published by Chen et al. (23). In 2009 concluded that Shenmen point is the most commonly used acupoint for auricular therapy for insomnia. The auricular acupoint MA-TF1 (Shen Men) is located in the triangular fossa and receives nerve supply through the cranium (vagus nerve) (72). However, no researcher updated and summarized this field in recent years.

Secondly, auricular acupoint therapy has the advantages of relatively simple operation, short operation time, and high feasibility, which significantly reduces the work burden of clinical nurses. And the side effect of auricular therapy is rare. However, two systematic reviews suggested that auricular acupuncture may cause bleeding or infectious diseases, and the adverse events of auricular therapy were local skin irritation, discomfort, tenderness at the sticking site, irritation or pain of adhesive tape, dizziness, etc., which were primarily mild, short-term and welltolerated. At present, no study has shown that the efficacy of auricular acupuncture has a more significant advantage over auricular pressing (73, 74). More studies are needed to determine the optimal plan of auricular therapy for treating diseases. As the highest level of evidence, the systematic evaluation concluded that auricular therapy might be beneficial for insomnia (75), but the evidence was plagued by significant limitations (76).

It is worth noting that there is considerable heterogeneity in the intervention measures of different TCM Nursing Techniques for insomnia, especially in the selection of acupoints and stimulation frequency, which makes it difficult to compare and integrate the intervention methods of various RCTs. Therefore, to ensure the repeatability and reliability of research results, study design and quality control need to be further strengthened. In addition, no high-quality systematic review or meta-analysis has been conducted in the recent five years to systematically report the intervention time and follow-up time of acupoint selection stimulation frequency. The purpose of the systematic evaluation is primarily to verify effectiveness and safety (57, 58). There is an urgent need for more high-quality, rigorously designed large-scale randomized controlled clinical trials with longer duration to confirm the efficacy of TCM Nursing Techniques in the treatment of insomnia (77). In particular, it is necessary to study further to select the acupoint frequency better and intervention time stimulated by TCM Nursing Techniques in the treatment of insomnia, analyze and select the most effective TCM nursing program and operation procedures for the intervention of insomnia, in order to meet the needs of evidence-based practice and high-level clinical research, and promote the international trend of TCM. In addition, large-scale prospective studies are expected to clarify the efficacy of TCM Nursing Techniques in comorbid insomnia, and the mechanisms of action of these techniques will depend on the future animal and large-scale clinical trials.

### Limitations and Strengths

First, the database used in this study was Web of Science Core Collection, which included articles of randomized controlled trials published in English. The major reason we were not choosing Chinese literature from databases in China such as CNKI/WanFang is the quality of publications from these databases are uneven parameters. 57 of the 146 articles we included were published by Chinese scholars, accounting for 39 percent, and some high quality literatures from Chinese were publicated in WOS. According to that, we thought the results we searched from WOS could reflect Chinese research in TCM nursing techniques for insomnia. Therefore, articles not published in this database and those not published in English are excluded, especially those published in Chinese. Second, in terms of citation, authors may tend to cite articles from the journals they wish to post, so there may be selection bias. Thirdly, there are only 146 articles published in English in the past 20 years, and the Web of Science database is constantly updated. Therefore, newly published articles may have an impact on this research. Make full use of the limited evidence and our research using CiteSpace software visualization analysis. Our results indicate that not only can highlight by far the most influential in TCM Nursing Techniques to treat insomnia of literature, but also help guide the research target, provide more reference information and basis for future research, and support to promote nursing research to the clinical practice of TCM the transition of the course.

## Conclusion

This study comprehensively and objectively analyzed the clinical studies of TCM Nursing Techniques for insomnia published in the Web of Science database and explored and analyzed the overall development trend of significant national and institutional cooperation in the past 18 years. The current research status shows that this field has excellent development prospects, but China and scholars still need to strengthen cooperative relationships. Hot issues and research frontiers are sleep quality, comorbid insomnia, and clinical trial design. By observing the different TCM Nursing Techniques for a variety of the cause of insomnia sleep quality improvement, further refine the evaluation index of sleep, and determine the most effective insomnia of TCM nursing plan and intervention operation rules, to explore the mechanism of action of insomnia of TCM nursing intervention technology, actively apply various new methods and new ideas will be insomnia treatment the important developing direction in the future.

## Data Availability Statement

The original contributions presented in the study are included in the article/Supplementary Material, further inquiries can be directed to the corresponding author/s.

## Author Contributions

JW contributed to organize the study, prepared datasets, conducted statistical analysis, and drafted the manuscript. YChe verified the overall reproducibility of results and other research outputs and critical review, and commentary or revision of the manuscript in the pre-publication stage. YChu implemented presentation of the published work and specifically visualization presentation. XZ oversight and leadership responsibility for the research activity planning and execution. XM contributed to the study design, prepared the data set, and revised the manuscript. XL involved in management and coordination responsibility for the research activity planning and execution. All authors read and approved the final manuscript.

## Funding

This study was supported by the National Natural Science Foundation of China (No. 81503382), the Key project of Beijing University of Chinese Medicine (2020-JYB-ZDGG-078), and the Vertical Science Development Foundation of BUCM (2019-ZXFZJJ-039).

## Conflict of Interest

The authors declare that the research was conducted in the absence of any commercial or financial relationships that could be construed as a potential conflict of interest.

## Publisher's Note

All claims expressed in this article are solely those of the authors and do not necessarily represent those of their affiliated organizations, or those of the publisher, the editors and the reviewers. Any product that may be evaluated in this article, or claim that may be made by its manufacturer, is not guaranteed or endorsed by the publisher.

## Abbreviations

TCM nursing: Traditional Chinese Medicine Nursing
RCTs: Randomized Controlled Trials.

## References

[1] Rosenberg RP Krystal AD. Diagnosing and treating insomnia in adults and older adults. J Clin Psychiatry. (2021) 82:EI20008AH5C. 10.4088/JCP.EI20008AH5C 34587376

[2] Dopheide JA. Insomnia overview: epidemiology, pathophysiology, diagnosis and monitoring, and nonpharmacologic therapy. Am J Manag Care. (2020) 26:S76–S84. 10.37765/ajmc.2020.42769 32282177

[3] Su L Lu Z. Interpretation of Chinese guideline for insomnia disorder diagnosis and its treatment in 2017. World Clinic Drugs. (2018) 39:217–22. 10.13683/j.wph.2018.04.001

[4] Chinese Society of Neurology. Guidelines for diagnosis and Treatment of Adult Insomnia in China 2017. Chinese J Neurol. (2018) 51:324–35. 10.3760/cma.j.issn.1006-7876.2018.05.002

[5] Li ZH Zhang PD Chen Q Gao X Chung VCH Shen D. Association of sleep and circadian patterns and genetic risk with incident type 2 diabetes: a large prospective population-based cohort study. Eur J Endocrinol. (2021) 185:765–74. 10.1530/EJE-21-0314 34524977

[6] Li X Zhou T Ma H Huang T Gao X Manson JE. Healthy sleep patterns and risk of incident arrhythmias. J Am Coll Cardiol. (2021) 78:1197–207. 10.1016/j.jacc.2021.07.023 34531019 PMC8454031

[7] Choi H Youn S Um YH Kim TW Ju G Lee HJ. Korean clinical practice guideline for the diagnosis and treatment of insomnia in adults. Psychiatry Investig. (2020) 17:1048–1059. 10.30773/pi.2020.0146 33198436 PMC7711116

[8] Edinger JD. Should we finally include quantitative criteria in our definition of insomnia? Sleep Med. (2016) 26:69–70. 10.1016/j.sleep.2016.03.005 27838238

[9] Castelnovo A Ferri R Punjabi NM Castronovo V Garbazza C Zucconi M. The paradox of paradoxical insomnia: a theoretical review towards a unifying evidence-based definition. Sleep Med Rev. (2019) 44:70–82. 10.1016/j.smrv.2018.12.007 30731262

[10] McArdle N Reynolds AC Hillman D Moses E Maddison K Melton P. Prevalence of common sleep disorders in a middle-aged community sample. J Clin Sleep Med. (2022) 22:47. 10.5664/jcsm.9886 35082023 PMC9163626

[11] Riemann D Baglioni C Bassetti C Bjorvatn B Dolenc Groselj L Ellis JG. European guideline for the diagnosis and treatment of insomnia. J Sleep Res. (2017) 26:675–700. 10.1111/jsr.12594 28875581

[12] Ree M Junge M Cunnington D. Australasian Sleep Association position statement regarding the use of psychological/behavioral treatments in the management of insomnia in adults. Sleep Med. (2017) 36:S43–S47. 10.1016/j.sleep.2017.03.017 28648226

[13] Liu S Zhang B. Interpretation of “guidelines for the diagnosis and treatment of insomnia disorder in China.” *Chinese J Contemp Neurol Neurosurg*. (2017) 17:633–8.

[14] Celikkayalar E Airaksinen M Kivelä SL Nieminen J Kleme J Puustinen J. Are older people aware of potential risks related to benzodiazepines they are taking and has anything changed in risk awareness over ten years? Patient Prefer Adher. (2021) 15:141–7. 10.2147/PPA.S280503 33536750 PMC7850572

[15] Airagnes G Pelissolo A Lavallée M Flament M Limosin F. Benzodiazepine misuse in the elderly: risk factors, consequences, and management. Curr Psychiatry Rep. (2016) 18:89. 10.1007/s11920-016-0727-9 27549604

[16] Mirchandaney R Barete R Asarnow LD. Moderators of cognitive behavioral treatment for insomnia on depression and anxiety outcomes. Curr Psychiatry Rep. (2022) 21:236. 10.1007/s11920-022-01326-3 35061137 PMC8948126

[17] Koffel E Bramoweth AD Ulmer CS. Increasing access to and utilization of cognitive behavioral therapy for insomnia (CBT-I): a narrative review. J Gen Intern Med. (2018) 33:955–62. 10.1007/s11606-018-4390-1 29619651 PMC5975165

[18] Yufang H Shiyuan W. Fundamentals of Traditional Chinese Medicine Nursing (Version 2). People's Medical Publishing House (2020).

[19] Wu Y Zhang Z Liu Y Shi G Ding X. The application effect of traditional chinese medicine nursing on general anesthesia combined with epidural anesthesia and electric resection for the treatment of bladder cancer and its influence on tumor markers. Evid Based Complement Alternat Med. (2022) 2022:7178711. 10.1155/2022/7178711 35075365 PMC8783706

[20] Guo FF Han D Wang JY Zhang ML Zhu JY. Effect of Fine Moxibustion on Sleep and Carotid Resistance Indexin Patients With Cervical Insomnia. Chinese Archives of TCM:1-9. Available online at: http://kns.cnki.net/kcms/detail/21.1546.R.20210511.0919.002.html (Chinese) (accsessed June 16, 2021).

[21] Li LC Xing HJ Liang Y Hu YH An X. Comparison of therapeutic effects between thermosensitive moxibustion and medication in the treatment of insomnia of liver-qi stagnation pattern. Acupuncture Res. (2018) 43:573–5. 10.13702/j.1000-0607.170765 30232866

[22] Meng F Gong WJ Liao YX Xu HW Wang X. Effect of auricular intradermal needling combined with erjian (HX6,7i) bloodletting on sleep quality and neuroendocrine level in patients with perimenopausal insomnia. Chinese Acupunct Moxibust. (2018) 38:575–9. 10.13703/j.0255-2930.2018.06.002 29971997

[23] Chen HY Shi Y Ng CS Chan SM Yung KK Zhang QL. Auricular acupuncture treatment for insomnia: a systematic review. J Altern Complement Med. (2007) 13:669–76. 10.1089/acm.2006.6400 17718650

[24] Chinese Medicine Academy of Chinese Medicine Insomnia Clinical practice Guidelines research group. Guidelines for clinical practice of Chinese medicine in insomnia (WHO/WPO). World J Sleep Med. (2016) 3:8–25.

[25] Insomnia cooperative group of state administration of traditional chinese medicine. Traditional chinese medicine diagnosis and treatment program of insomnia. World J Sleep Med. (2015) 2:14–8.

[26] National Administration of Traditional Chinese Medicine. Notice on Printing and Distributing Tcm Technical Manual for Nursing Staff. Chinese Medicine Administration Memo (2015). Availalble online at: http://www.satcm.gov.cn/yizhengsi/gongzuodongtai/2018-03-24/2691.html

[27] Chen C. CiteSpace II : Detecting and visualizing emerging trends[J]. J Am Soc Inform Sci Technol. (2006) 57:359–77. 10.1002/asi.2031731864046

[28] Yeh CH Suen LK Shen J Chien LC Liang Z Glick RM. Changes in sleep with auricular point acupressure for chronic low back pain. Behav Sleep Med. (2016) 14:279–94. 10.1080/15402002.2014.981820 26244591

[29] Yeung WF Ho FY Chung KF Zhang ZJ Yu BY Suen LK. Self-administered acupressure for insomnia disorder: a pilot randomized controlled trial. J Sleep Res. (2018) 27:220–31. 10.1111/jsr.12597 28884877

[30] Jiao Y Guo X Luo M Li S Liu A Zhao Y. Effect of transcutaneous vagus nerve stimulation at auricular concha for insomnia: a randomized clinical trial. Evid Based Complement Alternat Med. (2020) 2020:6049891. Erratum in: Evid Based Complement Alternat Med. (2020) 2020:2536573. 10.1155/2020/6049891 32831871 PMC7429019

[31] Ni J Wang F Wang B Zhou H Zhang N Shi H. Effectiveness and safety of auricular acupoint bloodletting in treatment of insomnia: an assessor-blinded pilot randomized controlled trial. J Tradit Chin Med. (2018) 38:763–768. 10.1016/S0254-6272(18)30916-6 32185994

[32] Kao CL Chen CH Lin WY Chiao YC Hsieh CL. Effect of auricular acupressure on peri- and early postmenopausal women with anxiety: a double-blinded, randomized, and controlled pilot study. Evid Based Complement Alternat Med. (2012) 2012:567639. 10.1155/2012/567639 22649475 PMC3358095

[33] Lai FC Chen IH Chen PJ Chen IJ Chien HW Yuan CF. Acupressure, sleep, and quality of life in institutionalized older adults: a randomized controlled trial. J Am Geriatr Soc. (2017) 65:e103–8. 10.1111/jgs.14729 28152177

[34] Cho MY Min ES Hur MH Lee MS. Effects of aromatherapy on the anxiety, vital signs, and sleep quality of percutaneous coronary intervention patients in intensive care units. Evid Based Complement Alternat Med. (2013) 2013:381381. 10.1155/2013/381381 23476690 PMC3588400

[35] Chen IH Yeh TP Yeh YC Chi MJ Chen MW Chou KR. Effects of acupressure on sleep quality and psychological distress in nursing home residents: a randomized controlled trial. J Am Med Dir Assoc. (2019) 20:822–829. 10.1016/j.jamda.2019.01.003 30797692

[36] Muz G Taşci S. Effect of aromatherapy via inhalation on the sleep quality and fatigue level in people undergoing hemodialysis. Appl Nurs Res. (2017) 37:28–35. 10.1016/j.apnr.2017.07.004 28985917

[37] Sentürk A Tekinsoy Kartin P. The effect of lavender oil application via inhalation pathway on hemodialysis patients' anxiety level and sleep quality. Holist Nurs Pract. (2018) 32:324–35. 10.1097/HNP.0000000000000292 30320657

[38] Carotenuto M Gallai B Parisi L Roccella M Esposito M. Acupressure therapy for insomnia in adolescents: a polysomnographic study. Neuropsychiatr Dis Treat. (2013) 9:157–62. 10.2147/NDT.S41892 23378768 PMC3559075

[39] Ju MS Lee S Bae I Hur MH Seong K Lee MS. Effects of aroma massage on home blood pressure, ambulatory blood pressure, and sleep quality in middle-aged women with hypertension. Evid Based Complement Alternat Med. (2013) 2013:403251. 10.1155/2013/403251 23431338 PMC3570933

[40] Mi-kyoung L Sunog L Ji-Ah S Mi-Eun K Myung-Haeng H. The effects of aromatherapy essential oil inhalation on stress, sleep quality and immunity in healthy adults: Randomized controlled trial. Euro J Integrat Med. (2017) 12:79–86. 10.1016/j.eujim.2017.04.009

[41] Cho EH Lee MY Hur MH. The effects of aromatherapy on intensive care unit patients' stress and sleep quality: a non-randomised controlled trial. Evid Based Complement Alternat Med. (2017) 2017:2856592. 10.1155/2017/2856592 29375641 PMC5742427

[42] Kuo HC Tsao Y Tu HY Dai ZH Creedy DK. Pilot randomized controlled trial of auricular point acupressure for sleep disturbances in women with ovarian cancer. Res Nurs Health. (2018) 41:469–79. 10.1002/nur.21885 30024027

[43] Yeh CH Chien LC Lin WC Bovbjerg DH van Londen GJ. Pilot randomized controlled trial of auricular point acupressure to manage symptom clusters of pain, fatigue, and disturbed sleep in breast cancer patients. Cancer Nurs. (2016) 39:402–10. 10.1097/NCC.0000000000000303 26390073

[44] Zhao B Li L Jiao Y Luo M Xu K Hong Y. Transcutaneous auricular vagus nerve stimulation in treating post-stroke insomnia monitored by resting-state fMRI: the first case report. Brain Stimul. (2019) 12:824–6. 10.1016/j.brs.2019.02.016 30871845

[45] Bergdahl L Broman JE Berman AH Haglund K von Knorring L Markström A. Sleep patterns in a randomized controlled trial of auricular acupuncture and cognitive behavioral therapy for insomnia. Complement Ther Clin Pract. (2017) 28:220–6. 10.1016/j.ctcp.2017.06.006 28779933

[46] Buysse DJ Reynolds CF 3rd Monk TH Berman SR Kupfer DJ. The Pittsburgh Sleep Quality Index: a new instrument for psychiatric practice and research. Psychiatry Res. (1989) 28:193–213. 10.1016/0165-1781(89)90047-4 2748771

[47] Karadag E Samancioglu S Ozden D Bakir E. Effects of aromatherapy on sleep quality and anxiety of patients. Nurs Crit Care. (2017) 22:105–12. 10.1111/nicc.12198 26211735

[48] Ancoli-Israel S Liu L Rissling M Natarajan L Neikrug AB Palmer BW. Sleep, fatigue, depression, and circadian activity rhythms in women with breast cancer before and after treatment: a 1-year longitudinal study. Support Care Cancer. (2014) 22:2535–45. 10.1007/s00520-014-2204-5 24733634 PMC4119484

[49] Chien LW Cheng SL Liu CF. The effect of lavender aromatherapy on autonomic nervous system in midlife women with insomnia. Evid Based Complement Alternat Med. (2012) 2012:740813. 10.1155/2012/740813 21869900 PMC3159017

[50] Afshar M Mohsenzadeh A Gilasi H Sadeghi-Gandomani H. The effects of guided imagery on state and trait anxiety and sleep quality among patients receiving hemodialysis: A randomized controlled trial. Complement Ther Med. (2018) 40:37–41. 10.1016/j.ctim.2018.07.006 30219466

[51] Bikmoradi A Seifi Z Poorolajal J Araghchian M Safiaryan R Oshvandi K. Effect of inhalation aromatherapy with lavender essential oil on stress and vital signs in patients undergoing coronary artery bypass surgery: a single-blinded randomized clinical trial. Complement Ther Med. (2015) 23:331–8. 10.1016/j.ctim.2014.12.001 26051567

[52] Arai YC Sakakima Y Kawanishi J Nishihara M Ito A Tawada Y. Auricular acupuncture at the “shenmen” and “point zero” points induced parasympathetic activation. Evid Based Complement Alternat Med. (2013) 2013:945063. 10.1155/2013/945063 23861718 PMC3687596

[53] Lillehei AS Halcon LL. A systematic review of the effect of inhaled essential oils on sleep. J Altern Complement Med. (2014) 20:441–51. 10.1089/acm.2013.0311 24720812

[54] Lan Y Wu X Tan HJ Wu N Xing JJ Wu FS. Auricular acupuncture with seed or pellet attachments for primary insomnia: a systematic review and meta-analysis. BMC Complement Altern Med. (2015) 15:103. 10.1186/s12906-015-0606-7 25886561 PMC4425871

[55] Afshar MK Moghadam ZB Taghizadeh Z Bekhradi R Montazeri A Mokhtari P. Lavender fragrance essential oil and the quality of sleep in postpartum women. Iran Red Crescent Med J. (2015) 17:e25880. 10.5812/ircmj.17(4)2015.25880 26023343 PMC4443384

[56] Kim ME Jun JH Hur MH. Effects of aromatherapy on sleep quality: a systematic review and meta-analysis. J Korean Acad Nurs. (2019) 49:655–76. 10.4040/jkan.2019.49.6.655 31932562

[57] Pei M Chen J Dong S Yang B Yang K Wei L. Auricular acupressure for insomnia in patients with maintenance hemodialysis: a systematic review and meta-analysis. Front Psychiatry. (2021) 12:576050. 10.3389/fpsyt.2021.576050 34349673 PMC8326797

[58] Zhao ZH Zhou Y Li WH Tang ZH Xia TW Han-Li. Auricular acupressure in patients with hypertension and insomnia: a systematic review and meta-analysis. Evid Based Complement Alternat Med. (2020). 2020:7279486. 10.1155/2020/7279486 PMC7317612 32655667

[59] Zou G Li Y Liu J Zhou S Xu J Qin L. Altered thalamic connectivity in insomnia disorder during wakefulness and sleep. Hum Brain Mapp. (2021) 42:259–70. 10.1002/hbm.25221 33048406 PMC7721231

[60] Jiao Y Han Y Li X Fang YG Liu ZH Zhou WN. Comparison of body, auricular, and abdominal acupuncture treatments for insomnia differentiated as internal harassment of phlegm-heat syndrome: an orthogonal design. Evid Based Complement Alternat Med. (2015) 2015:578972. 10.1155/2015/578972 26640498 PMC4657063

[61] Yeung WF Chung KF Poon MM Ho FY Zhang SP Zhang ZJ. Acupressure, reflexology, and auricular acupressure for insomnia: a systematic review of randomized controlled trials. Sleep Med. (2012) 13:971–84. 10.1016/j.sleep.2012.06.003 22841034

[62] Johnson EO Roth T Breslau N. The association of insomnia with anxiety disorders and depression: exploration of the direction of risk. J Psychiatr Res. (2006) 40:700–8. 10.1016/j.jpsychires.2006.07.008 16978649

[63] Taylor DJ Lichstein KL Durrence HH Reidel BW Bush AJ. Epidemiology of insomnia, depression, and anxiety. Sleep. (2005) 28:1457–64. 10.1093/sleep/28.11.1457 16335332

[64] Chen PJ Huang CL Weng SF Wu MP Ho CH Wang JJ. Relapse insomnia increases greater risk of anxiety and depression: evidence from a population-based 4-year cohort study. Sleep Med. (2017) 38:122–9. 10.1016/j.sleep.2017.07.016 29031746

[65] Wang X Gu J Liu J Hong H. Clinical evidence for acupressure with the improvement of sleep disorders in hemodialysis patients: A systematic review and meta-analysis. Complement Ther Clin Pract. (2020) 39:101151. 10.1016/j.ctcp.2020.101151 32379633

[66] Wang Z Hu X Su J Gao X Xu N Xing Y. The efficacy and safety stimulating a single acu-point Shenmen (HT 7) for managing insomnia: a systematic review of randomized controlled trials. Eur J Integrat Med. (2017) S1876382017301464. 10.1016/j.eujim.2017.08.010

[67] Blackburn L Achor S Allen B Bauchmire N Dunnington D Klisovic RB. The effect of aromatherapy on insomnia and other common symptoms among patients with acute leukemia. Oncol Nurs Forum. (2017) 44:E185–93. 10.1188/17.ONF.E185-E193 28640576

[68] Cheong MJ Kim S Kim JS Lee H Lyu YS Lee YR. A systematic literature review and meta-analysis of the clinical effects of aroma inhalation therapy on sleep problems. Medicine. (2021) 100:e24652. 10.1097/MD.0000000000024652 33655928 PMC7939222

[69] Tang Y Gong M Qin X Su H Wang Z Dong H. The therapeutic effect of aromatherapy on insomnia: a meta-analysis. J Affect Disord. (2021) 288:1–9. 10.1016/j.jad.2021.03.066 33839552

[70] Usichenko T Hacker H Lotze M. Transcutaneous auricular vagal nerve stimulation (taVNS) might be a mechanism behind the analgesic effects of auricular acupuncture. Brain Stimul. (2017) 10:1042–44. 10.1016/j.brs.2017.07.013 28803834

[71] Farmer AD Strzelczyk A Finisguerra A Gourine AV Gharabaghi A Hasan A. International consensus based review and recommendations for minimum reporting standards in research on transcutaneous vagus nerve stimulation (Version 2020). Front Hum Neurosci. (2021) 14:568051. 10.3389/fnhum.2020.568051 33854421 PMC8040977

[72] Usichenko TI Wetzel B Paul J Lysenyuk V Hahnenkamp K Fleckenstein J. Auricular acupoints with reduced skin resistance: detection in patients scheduled for hip arthroplasty. Med Acupunct. (2018) 30:308–312. 10.1089/acu.2018.1314 30671150 PMC6338557

[73] Tan JY Molassiotis A Wang T Suen LK. Adverse events of auricular therapy: a systematic review. Evid Based Complement Alternat Med. (2014) 2014:506758. 10.1155/2014/506758 25435890 PMC4241563

[74] Nielsen A Gereau S Tick H. Risks and safety of extended auricular therapy: a review of reviews and case reports of adverse events. Pain Med. (2020) 21:1276–1293. 10.1093/pm/pnz379 32430505

[75] Vieira A Reis AM Matos LC Machado J Moreira A. Does auriculotherapy have therapeutic effectiveness? an overview of systematic reviews. Complement Ther Clin Pract. (2018) 33:61–70. 10.1016/j.ctcp.2018.08.005 30396628

[76] Huang J Shen M Qin X Huang Y. Effectiveness of auricular acupuncture for insomnia: an overview of systematic reviews. Evid Based Complement Alternat Med. (2020) 2020:6920902. 10.1155/2020/6920902 32508952 PMC7244956

[77] Samara MT Huhn M Chiocchia V Schneider-Thoma J Wiegand M Salanti G. Efficacy, acceptability, and tolerability of all available treatments for insomnia in the elderly: a systematic review and network meta-analysis. Acta Psychiatr Scand. (2020) 142:6–17. 10.1111/acps.13201 32521042

