# Supplementary material for: Visualizing Research Trends and Identifying Hotspots of Traditional Chinese Medicine (TCM) Nursing Technology for Insomnia: A 18-Years Bibliometric Analysis of Web of Science Core Collection
Source: Front Neurol. 2022 Mar 31;13:816031. doi: 10.3389/fneur.2022.816031 (PMC9009417; doi:10.3389/fneur.2022.816031)
Supplement: Supplementary file 1 [file Data_Sheet_1.docx]

Supplementary Material

# Supplementary Tables

| Appendix 1 Researching strategies and results. | | |
| --- | --- | --- |
| Set | Search Query | Results |
| #31 | (#30) AND #5 | [177](https://www.webofscience.com/wos/woscc/summary/2bd19d20-1faa-4b25-b865-e09ee0cb6cda-0dddf7db/date-descending/1) |
| #30 | ((((#25) OR #26) OR #27) OR #28) OR #29 | [9,328](https://www.webofscience.com/wos/woscc/summary/f3103be4-b934-40c3-9a1a-c56fc468ed4b-0dddf566/date-descending/1) |
| #29 | ALL=(aromatherapy) | [1,363](https://www.webofscience.com/wos/woscc/summary/a589be5f-f45d-4343-9a7b-1a5dbc53d4e2-0ddde1eb/date-descending/1) |
| #28 | ALL=((Macerating Therapy) OR (Maceration)) | [3,677](https://www.webofscience.com/wos/woscc/summary/033810c6-7956-47a8-8445-d80934582653-0ddddc22/date-descending/1) |
| #27 | ALL=(Reflexotherapy) | [30](https://www.webofscience.com/wos/woscc/summary/7dc58e07-ef5a-4e25-95aa-84d059a3964f-0dddd9b2/date-descending/1) |
| #26 | ALL=((Retention Enema with Chinese Herb) OR (enema)) | [4,242](https://www.webofscience.com/wos/woscc/summary/1e1d8025-628c-48b1-9a5d-47fc42d0ea6c-0dddd5ff/date-descending/1) |
| #25 | ALL=((Chinese Medicine Ion Introduction Therapy) OR (TCD iontophoresis)) | [18](https://www.webofscience.com/wos/woscc/summary/db90cf32-3cfe-4d53-8661-16d10cabf0bc-0dddd3bc/date-descending/1) |
| #24 | (#23) AND #5 | [8](https://www.webofscience.com/wos/woscc/summary/59945354-c07f-43f5-9045-6904f0dc7aa0-0dddccaf/date-descending/1) |
| #23 | ((#20) OR #21) OR #22 | [1,725](https://www.webofscience.com/wos/woscc/summary/aac848a4-20e4-4491-999f-b8fec54cc974-0dddcacd/date-descending/1) |
| #22 | ALL=(Chinese medicine hot pressing compress) | 0 |
| #21 | ALL=((Fumigating and Steaming Therapy) OR (Steaming Therapy) OR (Fumigating Therapy) OR (TCM fumigation) OR (TCD sufforcating) OR (medical fumigation)) | [652](https://www.webofscience.com/wos/woscc/summary/9882f656-05a4-4de6-8e54-9c14bee13d5f-0dddc1c3/date-descending/1) |
| #20 | ALL=((Drug Smearing Therapy) OR (TCM coated)) | [1,074](https://www.webofscience.com/wos/woscc/summary/353aa372-cf50-4043-9481-7167c5b0fbfc-0dddbf6d/date-descending/1) |
| #19 | (#17) AND #5 | [47](https://www.webofscience.com/wos/woscc/summary/64327c62-727c-40d9-947f-5572bc5482f2-0dddba5a/date-descending/1) |
| #18 | (#16) AND #5 | [1,616](https://www.webofscience.com/wos/woscc/summary/b3a5a59a-669b-4773-910c-b48574dc451b-0ddd8d5e/date-descending/1) |
| #17 | ((((#7) OR #9) OR #10) OR #12) OR #14 | [23,132](https://www.webofscience.com/wos/woscc/summary/eafa98d9-eb5d-4091-8041-6a44491b85b8-0dddb698/date-descending/1) |
| #16 | (((#8) OR #11) OR #13) OR #15 | [175,472](https://www.webofscience.com/wos/woscc/summary/a9662093-7006-4f4f-a8b1-3a3037368520-0ddd8a2b/date-descending/1) |
| #15 | ALL=((Acupressure) OR (Tuina) OR (Massage)) | [9,212](https://www.webofscience.com/wos/woscc/summary/527a7c1d-5c51-4c81-a303-99eb063cfcc2-0ddd85fc/date-descending/1) |
| #14 | ALL=((Wet Compress Therapy) OR (Hot Compress Therapy) OR (Wet and hot compress of Chinese medicine)) | [56](https://www.webofscience.com/wos/woscc/summary/caf44e86-b962-4f3c-90ae-c8a02b64c8b6-0ddda4b3/date-descending/1) |
| #13 | ALL=((acupoint injection) OR (acupuncture point injection therapy) OR (point injection therapy)) | [5,106](https://www.webofscience.com/wos/woscc/summary/13ddd643-42da-483a-98fe-2b33f9972c45-0ddd8401/date-descending/1) |
| #12 | ALL=((cold compress therapy) OR (cold compress of Chinese medicine)) | [43](https://www.webofscience.com/wos/woscc/summary/8db2af6e-cbbd-4a57-9524-648efcc3b7ea-0ddda144/date-descending/1) |
| #11 | ALL=((Acupoint Application) OR (application therapy) OR (acupoint sticking therapy)) | [127,842](https://www.webofscience.com/wos/woscc/summary/59736975-bbd9-45b4-999d-0681c433da00-0ddd7ad1/date-descending/1) |
| #10 | ALL=((Foot Bath Therapy) OR (Washing with Herbal Bag) OR (Body Bathing Therapy) OR (wash and steep of chinese medicine)) | [132](https://www.webofscience.com/wos/woscc/summary/4b61465a-189b-409b-82bd-59cd132b089e-0ddd9e70/date-descending/1) |
| #9 | ALL=((Medical Wax Therapy) OR (Wax)) | [22,346](https://www.webofscience.com/wos/woscc/summary/bc317ae7-08a0-42fc-8563-caebfe5d4e49-0ddd9c62/date-descending/1) |
| #8 | ALL=((Cupping Therapy) OR (cupping) OR (cup)) | [34,413](https://www.webofscience.com/wos/woscc/summary/57216025-b98c-4638-9913-bd886a9e38a2-0ddd777d/date-descending/1) |
| #7 | ALL=(Scraping Therapy) | [564](https://www.webofscience.com/wos/woscc/summary/9247128e-5dcc-446a-8122-9675e9c71598-0ddd966d/date-descending/1) |
| #6 | (#4) AND #5 | [318](https://www.webofscience.com/wos/woscc/summary/531f57f1-347c-4ff2-9b24-2ab4e19fca5b-0dd739c5/date-descending/1) |
| #5 | ALL=((insomnia) OR (sleeplessness) OR (sleep initiation) OR (maintenance disorders) OR (disorders of initiating and maintaining sleep) OR (primary insomnia) OR (transient insomnia) OR (secondary insomnia) OR (insomnia disorder) OR (sleep initiation dysfunction) OR (quality of sleep) OR (sleep complaint) OR (sleep problem) OR (sleep disturbance) OR (sleep disorder)) | [134,984](https://www.webofscience.com/wos/woscc/summary/67084365-fcca-490b-a294-418a183242c6-0dd73089/date-descending/1) |
| #4 | ((#1) OR #2) OR #3 | [10,895](https://www.webofscience.com/wos/woscc/summary/7a6b0a3a-81dc-4c0b-8a12-2cbc447bcb2c-0dd735e9/date-descending/1) |
| #3 | ALL=((Auricular Seed Taping Therapy) OR (Auricular Acupuncture Therapy) OR (Auricular Acupressure) OR (Auricular Therapy) OR (ear point) OR (auricular acupoint) OR (ear acupuncture) OR (auricular point sticking) OR (semen vaccariae) OR (auricular acupuncture) OR (Auricular Acupoint Bloodletting) OR (Auricular Point Acupressure) OR (Auricular Magnetic Press Pellets) OR (Auricular Bleeding)) | [7,722](https://www.webofscience.com/wos/woscc/summary/d649512f-35c2-4196-b7ec-e64c22a75de2-0dd72944/date-descending/1) |
| #2 | ALL=(Moxibustion) | [1,343](https://www.webofscience.com/wos/woscc/summary/4a7a153d-f303-4428-8ac4-ee6a15abcf82-0dd724d7/date-descending/1) |
| #1 | ALL=((Appropriate health technology of TCM) OR (nursing of TCM) OR (integrated western and Chinese nursing) OR (TCM Characteristic Nursing) OR (TCM nursing) OR (TCM nursing)) | [1,928](https://www.webofscience.com/wos/woscc/summary/5bf728a0-8687-48a6-803d-e53377c6ff71-0dd7202e/date-descending/1) |

| Appendix 2 18 TCM nursing techniques stipulated in national standards, translation and retrieval strategies. | | |
| --- | --- | --- |
| TCM Nursing Technique | Theme Words Retrieval | Researching strategies |
| 刮痧技术 | Scraping Therapy | (Scraping Therapy) |
| 拔罐技术 | cupping | (Cupping Therapy) OR (cupping) OR (cup) |
| 麦粒灸技术 | Moxibustion | (Moxibustion) |
| 隔物灸技术 |  |  |
| 悬灸技术 |  |  |
| 蜡疗技术 | Medical Wax Therapy | (Medical Wax Therapy) OR (Wax) |
| 穴位敷贴技术 | Acupoint Application | (Acupoint Application) OR (application therapy) OR (acupoint sticking therapy) |
| 中药泡洗技术 | Foot Bath Therapy | (Foot Bath Therapy) OR (Washing with Herbal Bag) OR (Body Bathing Therapy) OR (wash and steep of chinese medicine) |
| 中药冷敷技术 | Cold compress therapy | (cold compress therapy) OR (cold compress of Chinese medicine) |
| 中药湿热敷技术 | Wet Compress Therapy | ((Wet Compress Therapy) OR (Hot Compress Therapy) OR (Wet and hot compress of Chinese medicine)) OR ((Macerating Therapy) OR (Maceration)) |
| 中药涂药技术 | Drug Smearing Therapy | (Drug Smearing Therapy) OR (Traditional Chinese medicine coated) |
| 中药熏蒸技术 | Fumigating and Steaming Therapy | ((Fumigating and Steaming Therapy) OR (Steaming Therapy) OR (Fumigating Therapy) OR (traditional Chinese medicine fumigation) OR (TCD sufforcating) OR (medical fumigation)) OR (aromatherapy) |
| 中药热熨敷技术 | Chinese medicine hot pressing compress | (Chinese medicine hot pressing compress) |
| 中药离子导入技术 | Chinese Medicine Ion Introduction Therapy | (Chinese Medicine Ion Introduction Therapy) OR (TCD iontophoresis) |
| 穴位注射技术 | Acupoint injection | (acupoint injection) OR (acupuncture point injection therapy) OR (point injection therapy) |
| 耳穴贴压技术 | Auricular therapy | (Auricular Seed Taping Therapy) OR (Auricular Acupuncture Therapy) OR (Auricular Acupressure) OR (Auricular Therapy) OR (ear point) OR (auricular acupoint) OR (ear acupuncture) OR (auricular point sticking) OR (semen vaccariae) OR (auricular acupuncture) OR (Auricular Acupoint Bloodletting) OR (Auricular Point Acupressure) OR (Auricular Magnetic Press Pellets) OR (Auricular Bleeding) |
| 经穴推拿技术 | Acupressure | ((Acupressure) OR (Tuina) OR (Massage)) OR (Reflexotherapy) |
| 中药灌肠技术 | Retention Enema with Chinese Herb | (Retention Enema with Chinese Herb) OR (enema) |
